# Fiber intake and risk of chronic obstructive pulmonary disease: A systematic review and dose response meta‐analysis

**DOI:** 10.1002/fsn3.3640

**Published:** 2023-08-23

**Authors:** Neda Valisoltani, Seyed Mojtaba Ghoreishy, Hossein Imani, Asma Rajabi Harsini, Mohammadreza Jowshan, Nikolaj Travica, Hamed Mohammadi

**Affiliations:** ^1^ Department of Clinical Nutrition, School of Nutritional Sciences and Dietetics Tehran University of Medical Sciences Tehran Iran; ^2^ Department of Nutrition, School of Public Health Iran University of Medical Sciences Tehran Iran; ^3^ Student Research Committee, School of Public Health Iran University of Medical Sciences Tehran Iran; ^4^ Food and Mood Centre, IMPACT‐The Institute for Mental and Physical Health and Clinical Translation, School of Medicine, Barwon Health Deakin University Geelong Victoria Australia

**Keywords:** COPD, dose–response and meta‐analysis, fiber, pulmonary disease

## Abstract

This systematic review and dose–response meta‐analysis examined the risk of chronic obstructive pulmonary disease (COPD) following dietary fiber intake. Relevant articles were retrieved from a variety of databases, including Scopus, Embase, and Medline, until March 2023. Spirometry was the most frequently used method for determining the presence of COPD. Based on the search, there were a total of 213,912 participants across five separate studies. Random effects model was used to combine the data and a dose–response analysis was further conducted. Five distinct cohort studies were identified. Based on highest versus lowest analysis, there was an inverse correlation between the intake of total fiber (RR, 0.72; 95%, CI: 0.64–0.80), cereal fiber (RR: 0.76, 95% CI 0.68, 0.86), and fruit fiber (RR: 0.75, 95% CI: 0.68, 0.83). Although this result was not significant for vegetable fiber (RR, 0.95; 95% CI, 0.84–1.07). Dose–response analysis revealed that daily increase of 10 g of total dietary fiber, cereal fiber, or fruit fiber reduced the risk of COPD by 26%, 21%, and 37%, respectively. The ROBINS‐E tool classified all cohort studies as having a moderate risk of bias. Total fiber, cereal fiber, and fruit fiber intake were found to have low credibility using the NutriGrade tool. Additionally, there is a lack of scientific evidence supporting the use of vegetable fiber. Larger, more comprehensive studies are required to confirm these findings.

## Graphical abstract

This systematic review and dose‐response meta‐analysis examined the risk of chronic obstructive pulmonary disease (COPD) following dietary fiber intake. Dose‐response analysis revealed that daily increase of 10 g of total dietary fiber, cereal fiber, or fruit fiber reduced the risk of COPD by 26%, 21%, and 37%, respectively. Consuming a high fiber diet was associated with a decreased risk of COPD. Larger, more comprehensive studies are required to confirm these findings.

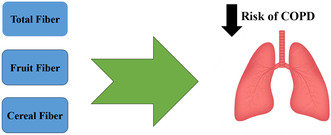

## 1 INTRODUCTION

Chronic obstructive pulmonary disease (COPD) is a significant global health problem characterized by persistent airflow obstruction and chronic inflammation that typically begins locally but progresses to systemic issues (e.g., losing weight, dyspnea, cardiovascular disease, etc.) (de Blasio et al., 2018; Kaluza et al., 2018). COPD has become the fourth leading cause of death in the United States due to an increase in global prevalence (Hurd, 2000). The World Health Organization predicts that as the prevalence of smoking increases, COPD will become the third leading cause of death in 2030 (Szmidt et al., 2020).

Smoking is the most common cause of COPD (Kaluza et al., 2018). Cigarette smoking causes inflammation and the production of reactive oxygen species (ROS) (Joshi et al., 2015). COPD can present itself in a variety of ways irrespective of the same smoking history between patients, adding to the complexity of the condition (Kaluza et al., 2018). Ethnicity, race, gender, respiratory disorders, genetics, occupation, and diet all play a role in the etiology of COPD (Buist, 1996). COPD places a great deal of strain on both the patient as well as society. The adverse effects associated with COPD include skeletal muscle weakness, sarcopenia, and an increased risk of lung diseases (such as pneumothorax and pneumonia) (de Blasio et al., 2018; Maddocks et al., 2015; Sekine et al., 2002). Recent research has demonstrated that anti‐inflammatory dietary components can significantly reduce the risk of COPD (Kaluza et al., 2018). In particular, vitamin and antioxidant‐rich fruits and vegetables can help prevent COPD (Kan et al., 2008). Further research outlines the decreased risk of developing certain diseases as a result of dietary fiber intake (Szmidt et al., 2020). Previous research has established a link between dietary patterns and lung function. Chronic conditions such as COPD can benefit significantly from the DASH diet, which is high in fruits, vegetables, and legumes (Ardestani et al., 2017). In contrast to this, consumption of dietary fiber has been linked to an increased risk of developing COPD, according to a Korean cohort study of 1832 cases (Jung et al., 2021). Pankaj joshi et al. discovered no correlation between daily fiber intake and the risk of developing COPD in a study of 325 COPD patients (Joshi et al., 2015). According to a review of three cohort studies involving over 63,000 middle‐aged Chinese men and women, over 120,000 American women and men, and over 46,000 Swedish men, increasing dietary fiber intake can reduce the risk of COPD by 39% (Butler et al., 2004; Kaluza et al., 2018; Seyedrezazadeh et al., 2019; Varraso et al., 2010).

However, the results generated from previous studies have not been aggregated and analyzed quantitatively. Dose–response relationships between fiber intake and COPD risk have not been established. The aim of the meta‐analysis and dose–response analysis was to determine whether there is a relationship between fiber consumption and risk of developing COPD, and whether this relationship may depend on the amount of consumed fiber. These findings could encourage the consumption of dietary fiber as a way of preventing COPD.

## 2 METHODS

### 2.1 Search strategy

PRISMA guidelines were followed for the design and execution of the meta‐analysis (Seyedrezazadeh et al., 2019). The review was registered on PROSPERO (CRD42021277692). Medline, Web of Science, and Scopus databases were searched until March 2023. Subject headings and keywords were used in the search strategy without language and date limitations. The key search terms included were “Dietary Fiber” OR cellulose OR cereals OR fiber OR whole grain) AND (“Pulmonary Disease” OR “chronic obstructive bronchitis” OR “chronic obstructive pulmonary disease” OR copd OR “chronic obstructive airway disease” OR Emphysema OR Bronchitis) (Table S1). The reference list of all relevant publications was reviewed to avoid missing any paper. All these steps were performed by two independent investigators (NV, SMG). Any disagreements were resolved by discussion or if necessary, by the third investigator (HM). Duplicate citations were then removed. The full text of related articles was obtained, in some cases through contacting the corresponding author.

### 2.2 Inclusion and exclusion criteria

There were two key inclusion criteria: (1) all prospective and retrospective cohort studies examining the association between dietary fiber consumption and the risk of COPD, and (2) studies that presented multivariable‐adjusted risk ratios, hazards and odds ratios, and 95% confidence intervals (CI) for COPD risk and dietary fiber content. Exclusion criteria included the following: (1) animal studies or other study designs such as reviews, case reports, or letters; (2) reporting duplicate data from other included studies; (3) failing to examine the primary outcome measure (risk of COPD); and (4) containing insufficient or unextractable data.

### 2.3 Data extraction

Two authors extracted the relevant data (NV and SMG). Any discrepancies in data were discussed with a third author (HM). Additionally, demographic characteristics such as age and gender were considered during the extraction process, as well as the methods and doses used to assess dietary fiber intake (method of COPD diagnosis assessment). For analyses using multiple adjustment models, results from the model using the largest number of covariates were included.

### 2.4 Quality assessment for individuals

Two reviewers (NV and SMG) assessed the risk of bias and the quality of data using the ROBINS‐E tool, evaluating multiple domains (Table S2): bias due to confounding, bias due to participant selection, bias due to exposure assessment, bias due to follow‐up misclassification, bias due to missing data, bias due to outcome measurement, and bias due to selective reporting. Bias was classified as low, moderate, serious, or critical across all of the domains. NutriGrade was used to quantify the degree to which total fiber, cereal fiber, fruit fiber, and vegetable fiber were associated with a decreased risk of COPD (Schwingshackl et al., 2016). The level of evidence certainty was calculated using the following scale: very low (0 to <4 points), low (4 to <6 points), moderate (6 to <8 points), or high (8 to 10 points) (Table S3).

### 2.5 Statistical analysis

The effect size was calculated using the RR and 95% CIs from cohort studies. When cohort study results were compared, the reported hazard ratios were consistent with the risk ratios. The reported HRs in cohort studies were equal to RRs (Symons & Moore, 2002). The ORs in cohort studies were considered equal to RR if the incidence rate in included studies was low (<10%) or ORs were between 0.5 and 2.5; otherwise, we converted OR to RR based on Zhang et al. method (Zhang & Yu, 1998).

Total fiber, cereal fiber, vegetable fiber, and fruit fiber intake in the highest and lowest categories were combined for our primary meta‐analysis. Summary RR and OR were calculated using a random‐effects model (Higgins et al., 2003). With the use of subgroup analyses, we looked for the sources of heterogeneity by taking into account factors such as gender differences, follow‐up duration, geographical region, and adjustment for main confounders. To test for publication bias, Egger and Begg's tests were used (Begg & Mazumdar, 1994; Egger et al., 1997). A sensitivity analysis was performed by removing one study at a time to determine the relative impact of each study on the overall estimate.

The linear dose–response analysis was conducted using Greenland et al.'s generalized least squares trend estimation method (Berlin et al., 1993; Orsini et al., 2006). We required a distribution of cases and participants or person years for this method, as well as adjusted effect sizes for each exposure category. Each RR or OR was based on the median fiber intake in that category. If exact medians were unavailable, we used the midpoint between the lower and upper boundaries as an approximation. When the highest and lowest categories were open‐ended, we assumed that open‐ ended categories had the same interval as the adjacent intervals. For studies that the exposures were defined as quantiles but did not report the numbers of participants, cases of COPD or person years in each category, we calculated these values by dividing the total number of participants, cases or person years by the number of categories (Jayedi et al., 2018).

Each study was pooled using a random effects model, and the pooled effect size was used in the meta‐analysis if the studies reported separately calculated effect sizes for smoking status. After estimating effect sizes for daily increases in total fiber, cereal, vegetable, and fruit fiber of one serving (10 g), RRs and ORs were calculated using a random effects model. If effect sizes were reported for exposure increments of one serving per day, we included them in the meta‐analysis. For studies that reported the effect size per specific increase in amount of exposure, we exponentiated the log effect size times the study specific intake of exposure to obtain the effect size for one additional serving of exposure.

According to Harrell, a non‐linear dose–response analysis was conducted using restricted cubic splines with knots at the 10%, 50%, and 90% percentiles of the distribution (Harre et al., 1988). The correlation within each category of published RRs was considered and the study specific estimates were combined by using a one stage linear mixed effects meta‐analysis (Crippa et al., 2019). Rather than requiring two stages as conventional methods, this technique estimates the study‐specific slope lines and then combines them to produce the overall average slope in a single step (Crippa et al., 2019; Greenland & Longnecker, 1992). Stata software was used to conduct all of our statistical analyses (version 14; StataCorp).

## 3 RESULTS

### 3.1 Study selection

**FIGURE 1:**
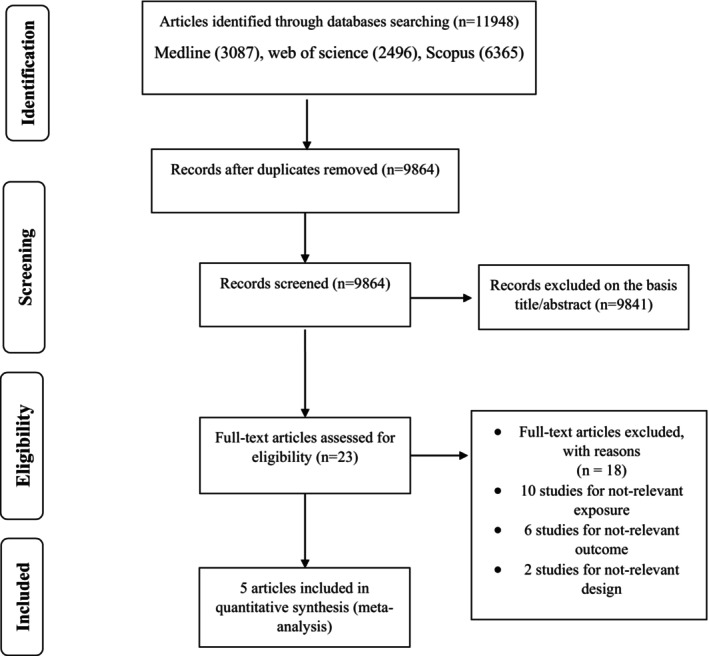
Literature search and review flow chart for selection of studies.

Out of 11,948 titles and abstracts, we identified 2084 duplicate studies, leaving only 9864 unique studies for the initial screening. Based on the inclusion criteria, the full texts of 23 studies were screened (Figure 1). A total of 18 studies were discarded for the reasons detailed in Table S4, which included irrelevant research (*n* = 10), lack of significant outcomes (*n* = 6), and lack of relevant experimental designs (*n* = 2). Finally, five cohort studies were eligible for inclusion into the meta‐analysis.

### 3.2 Characteristics of included studies

Table 1 summarizes the general characteristics of the included studies. Between 2008 and 2019, a total of 213,912 participants were included in the studies. All of the studies involved adults aged 30–79 years. Studies were conducted in the United States (Kan et al., 2008; Varraso et al., 2010), Sweden (Kaluza et al., 2018; Szmidt, et al., 2019) and South Korea (Joshi et al., 2015). Cohort studies included follow‐up periods ranging from 2 to 16 years. Food frequency questionnaires (FFQ) were used to determine the level of dietary fiber intake (Joshi et al., 2015; Kaluza et al., 2018; Kan et al., 2008; Szmidt et al., 2019; Varraso et al., 2010). In terms of exposure, all of the studies reported effect sizes for total fiber intake (Joshi et al., 2015; Kaluza et al., 2018; Kan et al., 2008; Szmidt et al., 2019; Varraso et al., 2010), four for cereal and vegetable fiber intake (Kaluza et al., 2018; Kan et al., 2008; Szmidt et al., 2019; Varraso et al., 2010) and three for fruit fiber intake (Kaluza et al., 2018; Szmidt et al., 2019; Varraso et al., 2010). One of the studies enrolled only women (Szmidt et al., 2019) and one enrolled only men (Kaluza et al., 2018), while the remainder were carried out on both sexes (Joshi et al., 2015; Kan et al., 2008; Varraso et al., 2010).

**TABLE 1:** Characteristics of included studies.

Author (year), country	Study design	Age^a^, gender	Follow up^b^	Total/case	Exposure type	Contrast	RR (95% CI), Highest versus Lowest	Exposure assessment (items)	Outcome assessment	Adjustments^c^
Kan et al. (2008), US	Cohort	44–66 Both	2 years	11,897/1753	Total fiber Cereal fiber Fruit fiber	C5 versus C1 C5 versus C1 C5 versus C1	0.8 (0.63–1.02) 0.79 (0.64–0.98) 0.81 (0.64–1.03)	FFQ	FEV1/FVC ratio < 0.7 and FEV1 < 80 percent	1, 2, 4, 5, 7, 15, 17, 20, 27, 42, 43, 44, 45, 46, 47, 48, 49, 50
Varraso et al. (2010), US	Cohort	30‐55 and 40–75 Both	16 years	111,580/832	Total fiber Cereal fiber Fruit fiber Vegetable fiber	C5 versus C1 C5 versus C1 C5 versus C1 C5 versus C1	0.67 (0.5–0.9) 0.77 (0.59–0.99) 0.77 (0.59–1.01) 0.92 (0.71–1.18)	FFQ	biennial questionnaire‐physician‐diagnosed	1, 2, 4, 9, 15, 17, 18, 27, 39, 40, 41, 42, 47
Joshi et al. (2015), Korea	Cohort	40–69 Both	5 years	10,038/325	Total fiber	C5 versus C1	0.92 (0.58–1.45)	FFQ	FEV1 and FVC were measured by spirometry	1, 2, 4, 8, 15, 17, 37, 38
Kaluza et al. (2018), Sweden	Cohort	45–79 Men	14 years	45,058/1982	Total fiber Cereal fiber Fruit fiber Vegetable fiber	C5 versus C1 C5 versus C1 C5 versus C1 C5 versus C1	0.63 (0.49–0.81) 0.66 (0.56–0.78) 0.71 (0.61–0.83) 0.89 (0.66–1.21)	FFQ	NR	1, 4, 5, 15, 16, 17, 18, 19, 47
Szmidt et al. (2020), Sweden	Cohort	50–75 Women	12 years	35,339/1557	Total fiber Cereal fiber Fruit fiber Vegetable fiber	C5 versus C1 C5 versus C1 C5 versus C1 C5 versus C1	0.71 (0.59–0.84) 0.84 (0.72–0.98) 0.77 (0.65–0.91) 0.97 (0.83–1.13)	FFQ	NR	1, 4, 5, 15, 16, 17, 18, 47

Abbreviations: C, category; FFQ, Food Frequency Questionnaire; Q, quintile; RR, risk ratio.

^a^ Presented as mean or range.

^b^ Number of years that individuals were followed up in the prospective cohort studies.

^c^ Adjustments: 1. Age, 2. Sex, 3. Race, 4. BMI, 5. Educational status or highest academic degree, 6. Marital status, 7. Occupational position, 8. Income, 9. Residence, 10. Size of residential area, 11. Wealth or household wealth, 12. Living alone, 13. Family arrangement, 14. Census track income data, 15. Tobacco or smoking, 16. Alcohol drinking, 17. Energy intake, 18. Physical activity level, 19. Fruit and vegetable and cereal fiber intake, 20. Dietary supplement use, 21. Cognition, 22. Depressed mood or depression, 23. Medical history, 24. Self‐rated health, 25. History of chronic disease, 26. Cardiovascular disease, 27. Diabetes mellitus, 28. Cancer, 29. Asthma or chronic bronchitis, 30. Osteo‐muscular disease, 31. Hyperlipidemia, 32. Number of drug treatment, 33. Postmenopausal hormone use, 34. Aspirin, 35. antihypertensive medications, 36. Lipid lowering medications, 37. Marriage status, 38. History of asthma and tuberculosis, 39. Physician visits, 40. Omega‐3 polyunsaturated fatty acid intake, 41. Cured meat intake, 42. Total fiber intake, 43. Height, 44. Square of height, 45. Study center, 46. Ethnicity, 47. Pack years of smoking, 48. Traffic density, 49. Glycemic index, 50. micronutrients.

### 3.3 Total fiber intake and risk of COPD

**FIGURE 2:**
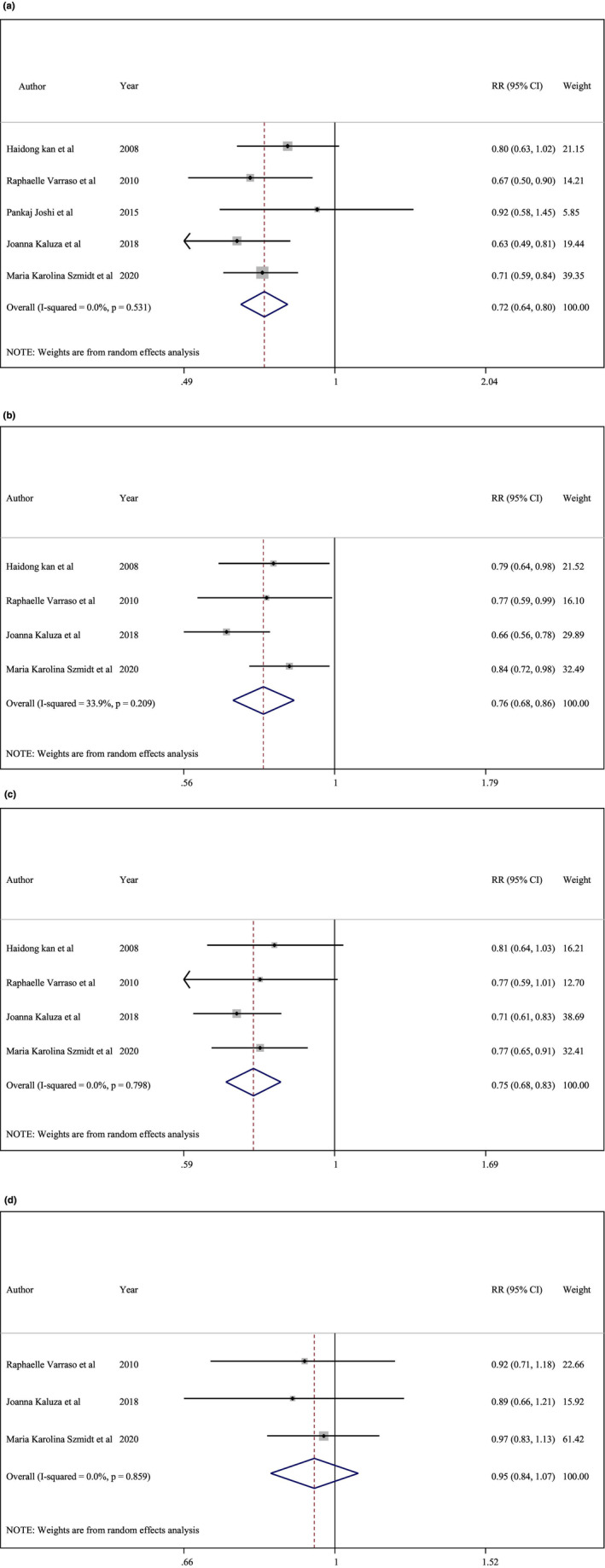
(a) Total fiber intake and risk of chronic obstructive pulmonary disease (COPD). (b) Cereal fiber intake and risk of COPD. (c) Fruit intake and risk of COPD. (d) Vegetable fiber intake and risk of COPD.

The association between dietary fiber intake and the risk of developing COPD was investigated using 5 cohort studies (Joshi et al., 2015; Kaluza et al., 2018; Kan et al., 2008; Szmidt et al., 2020; Varraso et al., 2010) involving a total of 213,912 participants, comprising of 6449 COPD cases. In regards to total fiber, those who ate more fiber had a lower risk of COPD (RR, 0.72; 95%, CI: 0.64–0.80; Figure 2a). There was no evidence of significant heterogeneity between studies, although the subgroup analysis was done to check the difference between the studies in terms of gender differences, follow‐up duration, geographical region and adjustment for main confounders. No significant difference was found between the mentioned subgroups (*I* ^2^ = 0%, *p* = .53, Table 2). There was no effect on the RR when each study was subjected to a sensitivity analysis. Begg and Egger's tests revealed no evidence of publication bias.

**TABLE 2:** Subgroup analysis to assess the total fiber intake and risk of chronic obstructive pulmonary disease.

Sub grouped by	No.	Weighted mean difference (95% CI)	*p*‐Value	*p*‐Heterogeneity	*I* ^2^ (%)	*p*‐Between subgroup heterogeneity
Sex						
Male	1	0.63(0.49, 0.81)	—	<.001	—	.443
Female	1	0.71(0.60, 0.85)	—	<.001	—	
Both	3	0.77(0.65, 0.91)	.46	.003	0%	
Follow‐up duration						
≥8 years	3	0.68(0.60, 0.77)	.743	<.001	0%	.130
<8 years	2	0.82(0.67, 1.02)	.597	.076	0%	
Geographical region						
US	2	0.74(0.62, 0.90)	.36	.002	0%	.608
Non‐US	3	0.70(0.61, 0.81)	.35	<.001	3.2%	
Adjustment for main confounders						
Yes	2	0.68(0.59, 0.79)	.44	<.001	0%	.306
No	3	0.77(0.65, 0.91)	.46	.003	0%	

**FIGURE 3:**
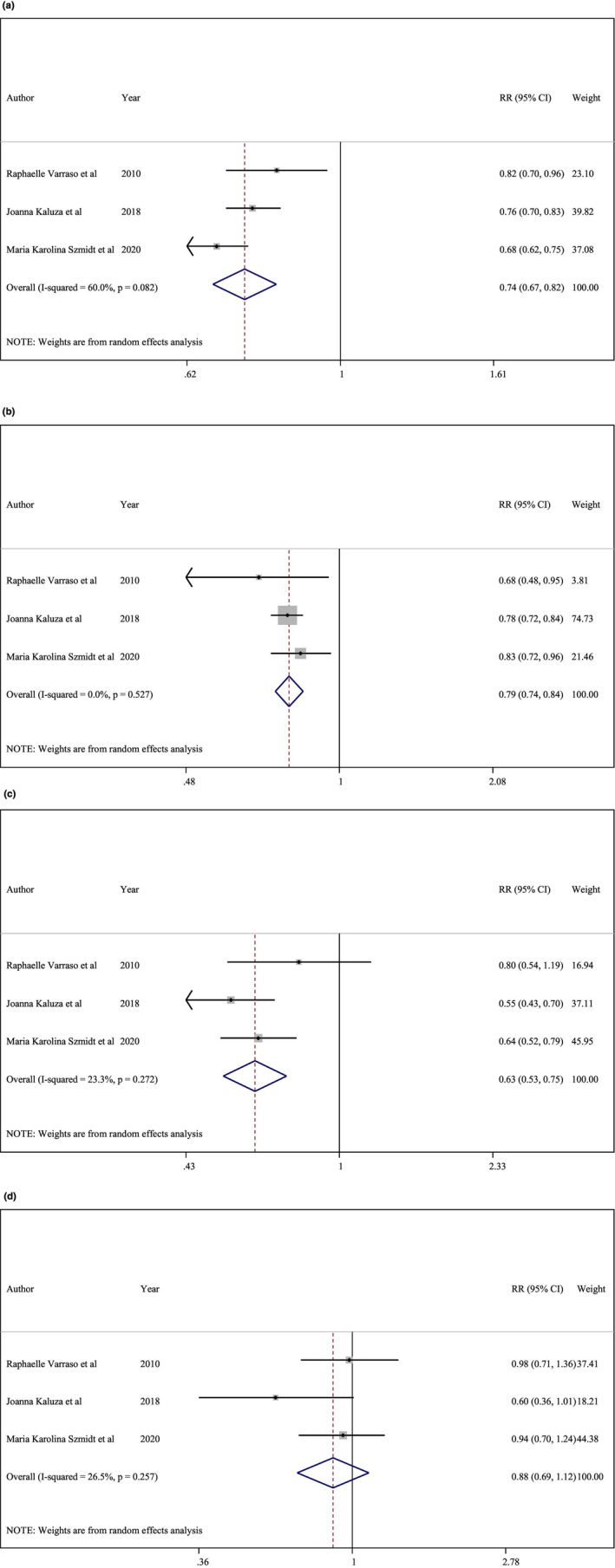
(a) Total fiber intake and risk of chronic obstructive pulmonary disease (COPD). (b) Cereal fiber intake and risk of COPD. (c) Fruit fiber intake and risk of COPD. (d) Vegetable fiber intake and risk of COPD.

**FIGURE 4:**
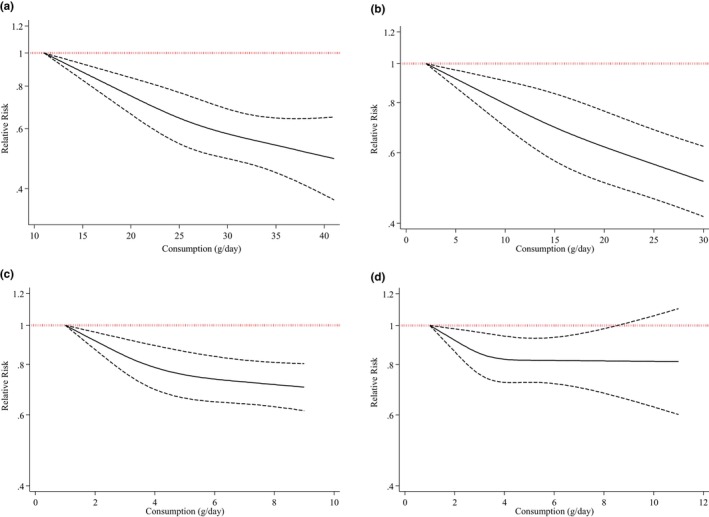
(a) Total fiber intake (*p*‐nonlinearity < .001, *n* = 3). (b) Cereal fiber intake (*p*‐nonlinearity < .001, *n* = 3). (c) Fruit fiber intake (*p*‐nonlinearity < .001, *n* = 3). (d) Vegetable fiber intake (*p*‐nonlinearity = .008; *n* = 3).

In three studies (Kaluza et al., 2018; Szmidt et al., 2020; Varraso et al., 2010), an increased intake of dietary fiber was associated with a 26% lower risk of COPD (RR, 0.74; 95% CI, 0.67–0.82; Figure 3a). There was a significant reduction in the risk of COPD following 12.5 g of dietary fiber intake (*p*‐nonlinearity < .001, *n* = 3; Figure 4a).

### 3.4 Cereal fiber intake and risk of COPD

The analysis of cereal fiber intake included over 203,874 individuals from four prospective cohort studies (Kaluza et al., 2018; Kan et al., 2008; Szmidt et al., 2020; Varraso et al., 2010) (total *n* = 203,874) with a total of 6142 cases. Using the highest and lowest levels of cereal fiber intake, it was discovered that the risk of COPD was greater in the highest category (RR: 0.76, 95% CI 0.68, 0.86; Figure 2b). There was a non‐significant heterogeneity between studies, but the subgroup analysis was performed to check the difference between the studies in terms of gender differences, follow‐up duration, geographical region and adjustment for main confounders. No significant difference was found between the mentioned subgroups (*I* ^2^ = 33.9%, *p* = .20, Table 3). After excluding all studies (one study at a time), the RR remained constant in a sensitivity analysis. Egger's (*p* = .98) and Begg's (*p* = .73) tests revealed no evidence of publication bias.

**TABLE 3:** Subgroup analysis to assess the cereal fiber intake and risk of chronic obstructive pulmonary disease.

Sub grouped by	No.	WMD (95% CI)	*p*‐Value	*p*‐Heterogeneity	*I* ^2^ (%)	*p*‐Between subgroup heterogeneity
Sex						
Male	1	0.66(0.56, 0.78)	—	<.001	—	.104
Female	1	0.84(0.72, 0.98)	—	.027	—	
Both	2	0.78(0.66, 0.92)	.88	.003	0%	
Follow‐up duration						
≥8 years	3	0.75(0.68, 0.86)	.20	<.001	43.9%	.700
<8 years	1	0.79(0.64, 0.98)	—	.030	—	
Geographical region						
US	2	0.78(0.66, 0.92)	.88	.003	0%	.693
Non‐US	2	0.75(0.59, 0.94)	.30	.015	0%	
Adjustment for main confounders						
Yes	2	0.75(0.59, 0.94)	.20	.015	0%	.693
No	2	0.78(0.66, 0.92)	.88	.003	0%	

We discovered a 21% reduction in the risk of COPD for every additional 10 g of cereal fiber consumed on a daily basis (Kaluza et al., 2018; Szmidt et al., 2020; Varraso et al., 2010) (RR, 0.79; 95% CI, 0.74–0.84; Figure 3b). This conclusion was reached following a dose–response analysis of three studies (Kaluza et al., 2018; Szmidt et al., 2020; Varraso et al., 2010). The non‐linear dose–response analysis revealed a significant reduction in the risk of COPD with a daily consumption of about 2.5 g and more of cereal fiber (*p*‐nonlinearity < .001, *n* = 3, Figure 4b).

### 3.5 Fruit fiber intake and risk of COPD

In four prospective cohort studies involving 203,877 participants, 6142 people were identified as having COPD (Kaluza et al., 2018; Kan et al., 2008; Szmidt et al., 2020; Varraso et al., 2010). According to the studies, a higher fruit fiber intake was associated with a lower risk of developing COPD (RR: 0.75, 95% CI: 0.68, 0.83; Figure 2c). The heterogeneity between studies was not statistically significant, There was a non‐significant heterogeneity between studies, but the subgroup analysis was conducted to check the difference between the studies in terms of gender differences, follow‐up duration, geographical region and adjustment for main confounders. No significant difference was found between the mentioned subgroups (*I* ^2^ = 0%, *p* = .79, Table 4). The RR was unaffected by the omission of individual studies, as determined by a sensitivity analysis. Using Egger's and Begg's test (*p* = .30 and *p* = .73), respectively), it was determined that there was no evidence of publication bias.

**TABLE 4:** Subgroup analysis to assess the fruit fiber intake and risk of chronic obstructive pulmonary disease.

Sub grouped by	No.	WMD (95% CI)	*p*‐Value	*p*‐Heterogeneity	*I* ^2^ (%)	*p*‐Between subgroup heterogeneity
Sex						
Male	1	0.71(0.61, 0.83)	—	<.001	—	.625
Female	1	0.77(0.65, 0.91)	—	.002	—	
Both	2	0.79(0.66, 0.95)	.78	.010	0	
Follow‐up duration						
≥8 years	3	0.74(0.67, 0.82)	.75	<.001	0	.506
<8 years	1	0.81(0.64, 1.03)	—	.083	—	
Geographical region						
US	2	0.79(0.66, 0.95)	.78	.010	0	.501
Non‐US	2	0.74(0.66, 0.83)	.48	<.001	0	
Adjustment for main confounders						
Yes	2	0.74(0.66, 0.83)	.48	<.001	0	.501
No	2	0.79(0.66, 0.95)	.78	.010	0	

A dose–response analysis of three studies (Kaluza et al., 2018; Szmidt et al., 2020; Varraso et al., 2010) discovered a significant association between 10 g of fruit fiber intake per day and COPD risk (RR, 0.63; 95% CI, 0.53–0.75; Figure 3c). Daily consumption of 1 g of fruit fiber significantly reduced the risk of COPD (*p*‐nonlinearity < .001, *n* = 3) (Figure 4c).

### 3.6 Vegetable fiber intake and risk of COPD

There were 4371 cases of COPD among the 191,977 participants across three prospective cohort studies (Kaluza et al., 2018; Szmidt et al., 2020; Varraso et al., 2010), which were used to investigate the relationship between dietary intake of vegetable fiber and the risk of developing COPD. There was no evidence of a link between vegetable fiber intake and a decreased risk of developing COPD (RR, 0.95; 95% CI, 0.84–1.07; Figure 2d). There was no significant heterogeneity between studies (*I* ^2^ = 0%, *p* = .85). According to a sensitivity analysis, the RR remained unchanged when each study was excluded individually. No evidence of publication bias was discovered using Egger's (*p* = .03) or Begg's (*p* = .29) tests.

Three studies were eligible for a dose–response analysis (Kaluza et al., 2018; Szmidt et al., 2020; Varraso et al., 2010). Consumption of 10 g of vegetable fiber per day had no effect on the risk of COPD (RR, 0.88; 95% CI, 0.69–1.12; Figure 3d). The nonlinear dose–response meta‐analysis discovered that the risk of COPD was reduced linearly up to 4 g of vegetable fiber per day and then plateaued (*p*‐nonlinearity = .008; *n* = 3, Figure 4d).

### 3.7 Quality assessment of included studies

According to ROBINS‐E, all cohort studies had a moderate risk of bias (Table S2). There was a lack of certainty in the evidence relating total fiber, cereal fiber, and fruit fiber based on NutriGrade results. Additionally, there was insufficient evidence to support vegetable fiber consumption (Table S3).

## 4 DISCUSSION

This novel systematic review and dose–response meta‐analysis examined the relationship between dietary fiber intake and the risk of chronic obstructive pulmonary disease (COPD). According to findings, dietary fiber consumption was shown to significantly reduce the risk of developing COPD in cohort studies. COPD risk was reduced by 26%, 21%, and 37% for every additional daily 10 g of total dietary fiber, cereal fiber, and fruit fiber, respectively. There was no correlation between a high vegetable fiber intake and a decreased risk of COPD. The reviewed studies revealed no evidence of significant heterogeneity.

Patients with COPD are impacted by its progression across multiple organs, often resulting in a myriad of complications. COPD is characterized by breathing difficulties, persistent coughs, and recurrent lower respiratory tract infections. It is recommended to combine non‐pharmacological and pharmaceutical treatments in order to manage the severity and prevent exacerbations of COPD, as well as to improve overall quality of life (Ostroff, 2021). Previous research has linked a healthy diet to a lower risk of lung disease, including COPD. So that, increased vegetable and fruit consumption may slow the progression of COPD in male smokers (0.833, 95% CI: 0.727–0.938) (Celik & Topcu, 2006). Butler et al. suggested that a fruit‐based, high‐fiber diet may aid in the relief of chronic respiratory symptoms (0.67, 95% CI: 0.53, 0.86) (Butler et al., 2004). A further study indicated that dietary fiber may be critical for lung health (Hanson et al., 2016).

The Dietary Approaches to Stop Hypertension (DASH) eating plan is a low‐glycemic index, low‐energy dense diet that has been suggested for lowering blood pressure. The DASH diet focuses on vegetables, fruits, and whole grains. It includes fat‐free or low‐fat dairy products, fish, poultry, beans, and nuts (Vollmer et al., 2001). Numerous whole food diets, including the DASH diet, have been extensively studied for their efficacy in treating lung diseases. Adherence to the DASH diet was found to be negatively correlated with COPD symptoms in an Iranian hospital‐based case–control study (Ardestani et al., 2017). The DASH diet involves the consumption of large amounts of fruits and vegetables, high in fiber as opposed to processed foods (Ardestani et al., 2017). In an epidemiological study, children that ate fresh fruits, salads, and green vegetables were found to have improved ventilator function, despite the fact that vegetables had a weaker effect then fruits (Van Duyn & Pivonka, 2000). In line with this, two large‐scale studies concluded that there was no correlation between the prevalence of respiratory symptoms and vegetable consumption (Butler et al., 2004; “Influence of dietary protein and carbohydrate on oxidative biotransformation of drugs in normal adults and children with asthma,” 1981). Due to the potential of recall bias caused by self‐reporting, the results from the Singapore cohort study may have been significantly skewed (Butler et al., 2004). Previous research has established that dietary fiber has a protective effect against chronic diseases. Numerous countries consume varying amounts of dietary fiber. Recent studies indicate that the average fiber intake in the United States is 15 g per day, significantly less than the recommended 25–30 g per day (Jones, 2004). Iranians consume more carbohydrates than any other country, but a recent epidemiological study discovered that they do not consume enough fiber, fruits, and vegetables to meet the recommended daily allowances (Bahreinian & Esmaillzadeh, 2012).

New hypotheses have been generated as a result of research into the relationship between dietary fiber intake and an increased risk of COPD. According to the hypothesis, increasing dietary fiber intake may help reduce the risk of developing COPD. The pathogenesis of COPD is associated with inflammation of the airways and oxidative stress. Thus, the possible mechanisms of fiber involve its anti‐inflammatory and/or antioxidant properties (Kan et al., 2008). Consumption of dietary fiber has been shown to reduce C‐reactive protein (CRP), a marker of systemic inflammation (King et al., 2005, 2007; Liu et al., 2002; Ma et al., 2006). Fiber modulatory properties such as delaying glucose uptake (Basu et al., 2006) or reducing lipid oxidation can control inflammation (King et al., 2005). The gut microbiota may produce anti‐inflammatory cytokines as a result of fiber interactions (Poullis et al., 2004). Fiber‐rich diets may theoretically help lower the risk of heart disease and other chronic diseases (Kan et al., 2008).

This review has several distinct strengths. Despite the small number of articles included in the review, the total number of combined participants (*n* = 213,912) made this a thorough analysis. Consumption of dietary fiber was associated with an increased risk of COPD in a nonlinear dose–response analysis using the most advanced statistical method.

There are a number of limitations to consider. Given the observational nature of included studies, there is no evidence of a cause‐and‐effect relationship. Due to all of the included studies being conducted in first world countries, additional research in developing countries is necessary to gain a better understanding of the observed results. Our study was hampered by the fact that different studies used varying COPD definitions and diagnostic criteria over time. Although a FEV1/FVC ratio of <70% is the most frequently used definition of COPD in our review, this does not account for all possible sources of variation in the disease's definition. As a result of all included studies using a food frequency questionnaire (FFQ), there may have been differences in the classification of dietary fiber intake between studies. Given these limitations, studies may have been more open to discovering a inverse correlation between dietary fiber intake and risk of COPD than a positive correlation.

## 5 CONCLUSION

According to this meta‐analysis of five cohort studies, the risk of developing COPD was shown to be reduced with the daily consumption of 10 grams of total dietary fiber, cereal fiber, and fruit fiber. Consumption of vegetable fiber had no discernible effect on the incidence of COPD. Given that the evidence supporting a protective effect of dietary fiber on the risk of COPD was of low quality, larger and longer follow‐up prospective cohort studies are necessary to corroborate our findings.

## AUTHOR CONTRIBUTIONS

**Neda Valisoltani:** Investigation (equal); methodology (equal); writing – original draft (equal). **Seyed Mojtaba Ghoreishy:** Formal analysis (equal). **Hossein Imani:** Project administration (equal). **Asma Rajabi Harsini:** Investigation (equal). **Mohammadreza Jowshan:** Writing – review and editing (equal). **Nikolaj Travica:** Writing – review and editing (equal). **Hamed Mohammadi:** Project administration (equal).

## FUNDING INFORMATION

This study was financially supported by a grant from School of Nutrition Sciences and Dietetics, Tehran University of Medical Sciences (TUMS), Tehran, Iran.

## CONFLICT OF INTEREST STATEMENT

The authors declared no conflicts of interest.

## CONSENT FOR PUBLICATION

The participants were all adults, and the data provided to the researchers contained no personal information about them.

## Supporting information


Tables S1–S4.

## Data Availability

The datasets used and analysed during the current study available from the corresponding author on reasonable request.
