# Supplementary material for: Fiber intake and risk of chronic obstructive pulmonary disease: A systematic review and dose response meta‐analysis
Source: Food Sci Nutr. 2023 Aug 23;11(11):6775–88. doi: 10.1002/fsn3.3640 (PMC10630824; doi:10.1002/fsn3.3640)
Supplement: Supplementary file 1 — Tables S1–S4. [file FSN3-11-6775-s001.docx]

| **Database** | **key terms and the queries** |
| --- | --- |
| PubMed  (3087) | #1 "Dietary Fiber"[MeSH Terms] OR "Cellulose"[MeSH Terms] OR "Lignin"[MeSH Terms] OR "Edible Grain"[MeSH Terms] OR "Whole Grains"[MeSH Terms] OR "Psyllium"[MeSH Terms] OR "Sterculia"[MeSH Terms] OR "Carbon Fiber"[MeSH Terms] OR "Glucans"[MeSH Terms] OR "Galactans"[MeSH Terms] OR "Fructans"[MeSH Terms] OR "Carrageenan"[MeSH Terms] OR "Inulin"[MeSH Terms] OR "Lactulose"[MeSH Terms] OR "Sorghum"[MeSH Terms] OR "Secale"[MeSH Terms] OR "Millets"[MeSH Terms] OR "Bread"[MeSH Terms] OR "Pectins"[MeSH Terms] OR "Acacia"[MeSH Terms] OR "Peas"[MeSH Terms] OR "Oligosaccharides"[MeSH Terms] OR "Resistant Starch"[MeSH Terms] OR "cellulose"[Title/Abstract] OR "Lignin"[Title/Abstract] OR "cereals"[Title/Abstract] OR "psyllium"[Title/Abstract] OR "fiber"[Title/Abstract] OR "fibre"[Title/Abstract] OR "polysaccharides"[Title/Abstract] OR "Metamucil"[Title/Abstract] OR "polymers"[Title/Abstract] OR "carbohydrate"[Title/Abstract] OR "fermentable"[Title/Abstract] OR "fructan"[Title/Abstract] OR "asteraceae"[Title/Abstract] OR "fructooligosaccharide"[Title/Abstract] OR "oligofructose"[Title/Abstract] OR "inulin"[Title/Abstract] OR "lactulose"[Title/Abstract] OR "whole grain"[Title/Abstract] OR "edible grain"[Title/Abstract] OR "wheat"[Title/Abstract] OR "Rice"[Title/Abstract] OR "maize"[Title/Abstract] OR "oat"[Title/Abstract] OR "barley"[Title/Abstract] OR "Corn"[Title/Abstract] OR "Rye"[Title/Abstract] OR "millet"[Title/Abstract] OR "sorghum"[Title/Abstract] OR "Bread"[Title/Abstract] OR "sweets"[Title/Abstract] OR "desserts"[Title/Abstract] OR "Pasta"[Title/Abstract] OR "muffin"[Title/Abstract] OR "biscuit"[Title/Abstract] OR "pancake"[Title/Abstract] OR "waffle"[Title/Abstract] OR "Pectin"[Title/Abstract] OR "gum"[Title/Abstract] OR "Acacia"[Title/Abstract] OR "pea"[Title/Abstract] OR "oligosaccharide"[Title/Abstract] OR "resistant starch"[Title/Abstract] OR "soy polysaccharides"[Title/Abstract]  #2 "Pulmonary Disease"[Title/Abstract] OR "chronic obstructive bronchitis"[Title/Abstract] OR "chronic obstructive pulmonary disease"[Title/Abstract] OR "copd"[Title/Abstract] OR "chronic obstructive airway disease"[Title/Abstract] OR "chronic obstructive lung disease"[Title/Abstract] OR "Emphysema"[Title/Abstract] OR "Bronchitis"[Title/Abstract] OR "Airway Obstruction"[Title/Abstract] OR "chronic bronchitis"[Title/Abstract] OR "pulmonary disease, chronic obstructive"[MeSH Terms] OR "lung diseases, obstructive"[MeSH Terms] OR "Asthma-Chronic Obstructive Pulmonary Disease Overlap Syndrome"[MeSH Terms] OR "Pulmonary Emphysema"[MeSH Terms] OR "Emphysema"[MeSH Terms] OR "Bronchitis"[MeSH Terms] OR "bronchitis, chronic"[MeSH Terms] OR "Airway Obstruction"[MeSH Terms]  #1 and #2 |
| Scopus  (6365) | #1 ( TITLE-ABS-KEY ( "cellulose" )  OR  TITLE-ABS-KEY ( "Lignin" )  OR  TITLE-ABS-KEY ( "cereals" )  OR  TITLE-ABS-KEY ( "psyllium" )  OR  TITLE-ABS-KEY ( "fiber" )  OR  TITLE-ABS-KEY ( "fibre" )  OR  TITLE-ABS-KEY ( "polysaccharides" )  OR  TITLE-ABS-KEY ( "Metamucil" )  OR  TITLE-ABS-KEY ( "polymers" )  OR  TITLE-ABS-KEY ( "carbohydrate" )  OR  TITLE-ABS-KEY ( "fermentable" )  OR  TITLE-ABS-KEY ( "fructan" )  OR  TITLE-ABS-KEY ( "asteraceae" )  OR  TITLE-ABS-KEY ( "fructooligosaccharide" )  OR  TITLE-ABS-KEY ( "oligofructose" )  OR  TITLE-ABS-KEY ( "inulin" )  OR  TITLE-ABS-KEY ( "lactulose" )  OR  TITLE-ABS-KEY ( "whole grain" )  OR  TITLE-ABS-KEY ( "edible grain" )  OR  TITLE-ABS-KEY ( "wheat" )  OR  TITLE-ABS-KEY ( "Rice" )  OR  TITLE-ABS-KEY ( "maize" )  OR  TITLE-ABS-KEY ( "Oat" )  OR  TITLE-ABS-KEY ( "barley" )  OR  TITLE-ABS-KEY ( "Corn" )  OR  TITLE-ABS-KEY ( "Rye" )  OR  TITLE-ABS-KEY ( "millet" )  OR  TITLE-ABS-KEY ( "sorghum" )  OR  TITLE-ABS-KEY ( "Bread" )  OR  TITLE-ABS-KEY ( "sweets" )  OR  TITLE-ABS-KEY ( "desserts" )  OR  TITLE-ABS-KEY ( "Pasta" )  OR  TITLE-ABS-KEY ( "muffin" )  OR  TITLE-ABS-KEY ( "biscuit" )  OR  TITLE-ABS-KEY ( "pancake" )  OR  TITLE-ABS-KEY ( "waffle" )  OR  TITLE-ABS-KEY ( "Pectin" )  OR  TITLE-ABS-KEY ( "gum" )  OR  TITLE-ABS-KEY ( "Acacia" )  OR  TITLE-ABS-KEY ( "pea" )  OR  TITLE-ABS-KEY ( "Oligosaccharide" )  OR  TITLE-ABS-KEY ( "resistant starch" )  OR  TITLE-ABS-KEY ( "soy polysaccharides" ) )  #2 ( TITLE-ABS-KEY ( "pulmonary disease" )  OR  TITLE-ABS-KEY ( "chronic obstructive bronchitis" )  OR  TITLE-ABS-KEY ( "chronic obstructive pulmonary disease" )  OR  TITLE-ABS-KEY ( "copd" )  OR  TITLE-ABS-KEY ( "chronic obstructive airway disease" )  OR  TITLE-ABS-KEY ( "chronic obstructive lung disease " )  OR  TITLE-ABS-KEY ( "emphysema" )  OR  TITLE-ABS-KEY ( "bronchitis" )  OR  TITLE-ABS-KEY ( "airway obstruction" )  OR  TITLE-ABS-KEY ( "chronic bronchitis" ) )  #1 and #2 |
| Web of science  (2496) | #1  **TS=** (cellulose) *OR* **TS=** (lignin) *OR* **TS=** (cereals) *OR* **TS=** (psyllium) *OR* **TS=** (fiber) *OR* **TS=** (fibre) *OR* **TS=** (polysaccharides) *OR* **TS=** (metamucil) *OR* **TS=** (polymers) *OR* **TS=** (carbohydrate) *OR* **TS=** (fermentable) *OR* **TS=** (fructan) *OR* **TS=** (asteraceae) *OR* **TS=** (fructooligosaccharide) *OR* **TS=** (oligofructose) *OR* **TS=** (inulin) *OR* **TS=** (lactulose) *OR* **TS=** (“whole grain”) *OR* **TS=** (“edible grain”) *OR* **TS=** (wheat) *OR* **TS=** (rice) *OR* **TS=** (maize) *OR* **TS=** (oat) *OR* **TS=** (barley) *OR* **TS=** (Corn) *OR* **TS=** (rye) *OR* **TS=** (millet) *OR* **TS=** (sorghum) *OR* **TS=** (bread) *OR* **TS=** (sweets) *OR* **TS=** (desserts) *OR* **TS=** (pasta) *OR* **TS=** (muffin) *OR* **TS=** (biscuit) *OR* **TS=** (pancake) *OR* **TS=** (waffle) *OR* **TS=** (pectin) *OR* **TS=** (gum) *OR* **TS=** (acacia) *OR* **TS=** (pea) *OR* **TS=** (“resistant starch”) *OR* **TS=** (“soy polysaccharides”)  #2  **TS=** (“pulmonary disease”) *OR* **TS =** (“chronic obstructive bronchitis”) *OR* **TS =** (“chronic obstructive pulmonary disease”) *OR* **TS =** (copd) *OR* **TS =** (“chronic obstructive airway disease”) *OR* **TS =** (“chronic obstructive lung disease”) *OR* **TS =** (emphysema) *OR* **TS =** (bronchitis) *OR* **TS =** (“airway obstruction”) *OR* **TS =** (“chronic bronchitis”)  #1 and #2 |

**Supplementary Table 1.** Search strategies including the key terms and the queries for each database.

**Supplementary Table 2:** ROBINS-E judgement for each domain and overall

| **Study** | **Bias due to confounding** | **Bias due to selection of participants** | **Bias due to exposure assessment** | **Bias due to misclassification during follow-up** | **Bias due to missing data** | **Bias due to measurement of the outcome** | **Bias due to selective reporting of the results** | **Overall judgement** |
| --- | --- | --- | --- | --- | --- | --- | --- | --- |
| Haidong kan et al 2008 | Moderate | Moderate | Moderate | Moderate | Moderate | Moderate | Low | Moderate |
| Raphaelle Varraso et al 2010 | Moderate | Moderate | Moderate | Moderate | Moderate | Moderate | Low | Moderate |
| Pankaj Joshi et al 2015 | Moderate | Moderate | Moderate | Moderate | Moderate | Moderate | Low | Moderate |
| Joanna Kaluza et al 2018 | Moderate | Moderate | Moderate | Moderate | No information | Moderate | Low | Moderate |
| Maria Karolina Szmidt et al 2020 | Moderate | Moderate | Moderate | Moderate | Moderate | Moderate | Low | Moderate |

**Supplementary Table 3**. Scoring for the different components of NutriGrade for each outcome.

| **Exposure** | **Outcome** | **Risk of bias**^1^ | **Precision**^2^ | **Heterogeneity**^3^ | **Directness**^4^ | **Publication bias**^5^ | **Funding bias**^6^ | **Effect size**^7^ | **Dose-response**^8^ | **Sum** | **NutriGrade** |
| --- | --- | --- | --- | --- | --- | --- | --- | --- | --- | --- | --- |
| Total fiber | COPD | 1 | 0 | 0.5 | 0 | 0 | 1 | 1 | 1 | 4.5 | Low |
| Fruits fiber | COPD | 1 | 0 | 0.5 | 0 | 0 | 1 | 1 | 1 | 4.5 | Low |
| Vegetables fiber | COPD | 1 | 0 | 0.5 | 0 | 0 | 1 | 0 | 1 | 3.5 | Very low |
| Cereal fiber | COPD | 1 | 0 | 0.5 | 0 | 0 | 1 | 1 | 1 | 4.5 | Low |
| ^1^ Modifications of original NutriGrade-scoring system: Study quality Newcastle Ottawa Scale: ≥8 (2 points); ≥7-<8 (1.5 points), ≥6-<7(1 point).  ^2^ >5000 cases and the 95%CI excludes the null value (1 point), <5000 cases or >5000 cases but 95%CI includes the null value (0 point).  ^3^ 0 to 1 point on the basis of *I*^2^.  ^4^ No important differences in the population or hard clinical outcomes (1 point), important differences in the population (0 point).  ^5^ <5 studies or sever evidence of bias or publication bias not assessed (0 point), no evidence of bias for 5-9 studies or moderate evidence of bias for ≥10 studies (0.5 point), no evidence of bias for ≥10 studies (1 point).  ^6^ Funded by academic institutions or research institutions (1 point), funded by private institutions, foundations, or nongovernmental organizations (0.5 point), Industry funding or conflict of interest (0 point).  ^7^ No effect (RR: 0.80–1.20) when comparing the highest vs. lowest category (0 point), moderate effect size (RR: <0.80–0.50 and >1.20–2, and corresponding test is statistically significant) when comparing the highest vs. lowest category (1 point), large effect size (RR: <0.50 and >2.00, and corresponding test is statistically significant) when comparing the highest vs. lowest category (2 point).  ^8^ No dose-response analysis or dose-response analysis with corresponding statistical test no significant (0 point), significant linear or nonlinear dose-response relationship (1 point). | | | | | | | | | | | |

| **Supplemental Table 4**: Reason for exclusion of retrieved articles; fiber and chronic obstructive pulmonary disease | |
| --- | --- |
| **References** | **Reason for exclusion** |
| 1. Hain J. Impact of Dietary Fiber and Omega6/3 Intake on COPD Outcomes in US Adults. | not-relevant exposure |
| 1. Jung YJ, Lee SH, Chang JH, Lee HS, Kang EH, Lee SW. The Impact of Changes in the Intake of Fiber and Antioxidants on the Development of Chronic Obstructive Pulmonary Disease. Nutrients. 2021 Feb;13(2):580. | not-relevant exposure |
| 1. Ardestani ME, Onvani S, Esmailzadeh A, Feizi A, Azadbakht L. Adherence to Dietary Approaches to Stop Hypertension (DASH) dietary pattern in relation to chronic obstructive pulmonary disease (COPD): a case–control study. Journal of the American College of Nutrition. 2017 Oct 3;36(7):549-55. | not-relevant design |
| 1. Hanson C, Lyden E, Rennard S, Mannino DM, Rutten EP, Hopkins R, Young R. The relationship between dietary fiber intake and lung function in the National Health and Nutrition Examination Surveys. Annals of the American Thoracic Society. 2016 May;13(5):643-50.‏ | not-relevant outcome |
| 1. Kim T, Choi H, Kim J. Association Between Dietary Nutrient Intake and Chronic Obstructive Pulmonary Disease Severity: A Nationwide Population-Based Representative Sample. COPD: Journal of Chronic Obstructive Pulmonary Disease. 2020 Jan 2;17(1):49-58. | not-relevant exposure |
| 1. McKeever TM, Lewis SA, Cassano PA, Ocke M, Burney P, Britton J, Smit HA. Patterns of dietary intake and relation to respiratory disease, forced expiratory volume in 1 s, and decline in 5-y forced expiratory volume. The American journal of clinical nutrition. 2010 Aug 1;92(2):408-15. | not-relevant exposure |
| 1. Hirayama F, Lee AH, Binns CW, Zhao Y, Hiramatsu T, Tanikawa Y, Nishimura K, Taniguchi H. Do vegetables and fruits reduce the risk of chronic obstructive pulmonary disease? A case–control study in Japan. Preventive medicine. 2009 Aug 1;49(2-3):184-9. | not-relevant design |
| 1. ERBLAND M. Chronic Obstructive Pulmonary Disease and Carbohydrate Feedings. Annals of internal medicine. 1986 Jun 1;104(6):889-90. | not-relevant exposure |
| 1. Tabak C, Smit HA, Heederik D, Ocke MC, Kromhout D. Diet and chronic obstructive pulmonary disease: independent beneficial effects of fruits, whole grains, and alcohol (the MORGEN study). Clinical & Experimental Allergy. 2001 May;31(5):747-55. | not-relevant exposure |
| 1. Butler LM, Kan H, London SJ. Dietary fiber prevents both morbidity and mortality from respiratory disease. Archives of internal medicine. 2011 Jun 27;171(12):1123-. | not-relevant outcome |
| 1. Young RP, Hopkins RJ. High dietary fiber lowers systemic inflammation: potential utility in COPD and lung cancer. The American journal of medicine. 2014 Aug 1;127(8):e13. | not-relevant outcome |
| 1. Kaluza J, Harris HR, Linden A, Wolk A. Long-term consumption of fruits and vegetables and risk of chronic obstructive pulmonary disease: a prospective cohort study of women. International journal of epidemiology. 2018 Dec 1;47(6):1897-909. | not-relevant exposure |
| 1. Budden K, Shukla S, Rehman FS, Donvan C, Bowerman K, Dennis P, Hugenholtz P, Hansbro P. Dietary fibre elevates SCFAS and protects against experimental COPD. Respirology. 2019 Jan 1;24:123-. | not-relevant outcome |
| 1. Jang YO, Kim OH, Kim SJ, Lee SH, Yun S, Lim SE, Yoo HJ, Shin Y, Lee SW. High-fiber diets attenuate emphysema development via modulation of gut microbiota and metabolism. Scientific reports. 2021 Mar 26;11(1):1-1. | not-relevant outcome |
| 1. Vaughan A, Collins P, Budden K, Bowman R, Fong K, Hansbro P, Yang I. DIETARY SUPPLEMENTATION WITH INULIN REDUCES AIRWAY INFLAMMATION IN COPD: A PILOT STUDY. InRESPIROLOGY 2021 Apr 1 (Vol. 26, pp. 121-121). 111 RIVER ST, HOBOKEN 07030-5774, NJ USA: WILEY. | not-relevant exposure |
| 1. Malmir H, Onvani S, Ardestani ME, Feizi A, Azadbakht L, Esmaillzadeh A. Adherence to Low Carbohydrate Diet in Relation to Chronic Obstructive Pulmonary Disease. Frontiers in Nutrition. 2021;8. | not-relevant exposure |
| 1. Hedhli A, Slim A, Mjid M, Ouachi Y, Kacem M, Merai S, Toujani S, Dhahri B. Nutritional status and dietary intake in patients with chonic obstructive pulmonary disease. Revue des Maladies Respiratoires. 2021 Jun 3. | not-relevant exposure |
| 1. Saeed MA, Gribben KC, Alam M, Lyden ER, Hanson CK, LeVan TD. Association of dietary fiber on asthma, respiratory symptoms, and inflammation in the adult national health and nutrition examination survey population. Annals of the American Thoracic Society. 2020 Sep;17(9):1062-8. | not-relevant outcome |
